# Eight-Week Low-Intensity Squat Training at Slow Speed Simultaneously Improves Knee and Hip Flexion and Extension Strength

**DOI:** 10.3389/fphys.2020.00893

**Published:** 2020-07-24

**Authors:** Ryota Akagi, Shinya Sato, Naoya Hirata, Naoto Imaizumi, Hiroki Tanimoto, Ryosuke Ando, Ryoichi Ema, Kosuke Hirata

**Affiliations:** ^1^College of Systems Engineering and Science, Shibaura Institute of Technology, Saitama, Japan; ^2^Graduate School of Engineering and Science, Shibaura Institute of Technology, Saitama, Japan; ^3^Graduate School of Health Management, Keio University, Fujisawa, Japan; ^4^Department of Sports Research, Japan Institute of Sports Sciences, Tokyo, Japan; ^5^School of Management, Shizuoka Sangyo University, Iwata, Japan; ^6^Research Fellow of Japanese Society for the Promotion of Science, Tokyo, Japan

**Keywords:** muscle strength, muscle size, magnetic resonance imaging, electromyography, voluntary activation, low-intensity, slow speed

## Abstract

Considering that the squat exercise requires flexion and extension of the knee and hip joints, a resistance training program based on squat exercises should efficiently increase the flexion and extension strength of both the knee and hip. To our knowledge, however, no study has simultaneously investigated the effects of squat training on both flexion and extension strength in both the knee and hip. Low-intensity squat exercises at slow speeds can be expected to effectively and safely improve knee and hip flexion and extension strength in a wide range of individuals. This study aimed to clarify whether knee and hip flexion and extension strength improved after an 8-week low-intensity squat training program at slow speed. Twenty-four untrained young men were randomly assigned to a training or control group. Participants in the training group performed 40% one-repetition maximum parallel squats at slow speed (4 s for concentric/eccentric actions), 3 days per week for 8 weeks. Before and after the intervention, isometric peak torque of the knee and hip flexors and extensors during maximal voluntary contraction (MVC) was determined. For the knee flexors and extensors, muscle volume was also measured. There were significant training-induced increases in peak torque (*P* < 0.05). The training effects on knee and hip extension torque (effect size = 0.36–0.38) were higher than those on knee and hip flexion torque (effect size = 0.09–0.13). The squat training used here increased both knee and hip flexion and extension strength, but the training effects on the flexion strength were less than those on the extension strength. Regarding the knee extensors, a significant training-related increase in muscle volume was found (*P* < 0.05) without neuromuscular adaptations. In addition, there were significant correlations between the training-induced increases in muscle volume and peak torque of KE. These results suggest that muscle hypertrophy may be responsible for increased muscle strength of the knee extensors after an 8-week low-intensity squat training program at slow speed.

## Introduction

Muscle strength is one of the fundamental parameters producing human movement, and knee and hip flexion and extension strength play important roles in our daily activities, exercise, and sports (De Ste Croix et al., 2003; Bezodis et al., 2008; Tseng et al., 2016; Ema et al., 2018b). Resistance training has a beneficial impact on muscle strength (Erskine et al., 2010; Unhjem et al., 2015; Ema et al., 2018a), and is popular not only among athletes but also the general population. Considering that the squat exercise requires flexion and extension of the knee and hip joints, respectively, a resistance training program based on squat exercises may efficiently increase the flexion and extension strength of both the knee and hip. To our knowledge, however, no study has simultaneously investigated the effects of squat training on both flexion and extension strength in both the knee and hip. Therefore, it is important to address this challenge.

A previous study investigated the effects of 50% one-repetition maximum (1RM) squat training at two different speeds for 8 weeks (fast: 1 s and slow: 3 s for concentric/eccentric actions) on isometric knee/hip extension strength but not on knee/hip flexion strength (Usui et al., 2016). In that study, hip extension strength was significantly increased after the training at the slow speed but not fast speed. In addition, although no clear improvement in knee extension strength was found, the training at slow speed significantly increased the thicknesses of some of the knee extensors [KE; the rectus femoris and vastus intermedius muscles (RF and VI)] (Usui et al., 2016). Muscle size is a major determinant of muscle strength (Fukunaga et al., 2001; Akagi et al., 2009, 2012) although the question that remains is whether exercise-induced changes in muscle size contribute to muscle strength (Loenneke et al., 2019). Therefore, there is a possibility that squat training increases not only hip extension strength but also knee extension strength when the speed of concentric/eccentric actions is slower than 3 s and/or the intensity is changed from 50% 1RM. Moreover, given that the hamstrings, except for the short head of the biceps femoris muscle (BF), contribute to both hip extension and knee flexion (Frigo et al., 2010; Schache et al., 2010), and RF contributes to both knee extension and hip flexion (Ema et al., 2018a, b), such training should increase both knee and hip flexion strength.

In a previous study that conducted a systematic review of the current literature and a meta-analysis to compare changes in strength and hypertrophy between ≤ 60% 1RM vs. > 60% 1RM resistance training protocols (Schoenfeld et al., 2017), there were no significant differences in the training-induced increase in isometric strength and muscle size between the conditions. In addition, ∼50% 1RM resistance training at a slow speed (3 s for concentric/eccentric actions) has been shown to be useful for gaining muscle strength and size with low mechanical stress, estimated by systolic blood pressure compared with ∼80% 1RM resistance training at a fast speed (1 s for concentric/eccentric actions) (Tanimoto and Ishii, 2006). Considering these findings and the possibility described in the previous paragraph, low-intensity squat training at slow speed (> 3 s) can be expected to effectively and safely improve knee and hip flexion and extension strength for a wide range of individuals. In accordance with a systematic review regarding training intensity (Raymond et al., 2013), < 50% 1RM is categorized as the lowest intensity (named “low-intensity”). Hence, we hypothesized that knee and hip flexion and extension strength improve after low-intensity squat training at slow speed. The purpose of this study was to test this hypothesis using an 8-week low-intensity squat training at slow speed.

## Materials and Methods

### Participants

After providing written informed consent, 24 healthy young men who had not performed lower body resistance training for at least 1 year voluntarily participated in this study. They were randomly assigned to a training group [*n* = 12; age, 21 ± 2 year; height, 173.9 ± 5.1 cm; body mass, 64.2 ± 6.5 kg; mean ± standard deviation (SD)] or control group (*n* = 12; age, 22 ± 1 year; height, 170.3 ± 3.7 cm; body mass, 64.8 ± 12.6 kg). There were no significant differences in age, body height or body mass between the groups. None of the participants had a previous or current knee or hip injury. This study was approved by the Ethics Committee of the Shibaura Institute of Technology and conducted according to the Declaration of Helsinki.

### Experimental Procedures

**FIGURE 1:**
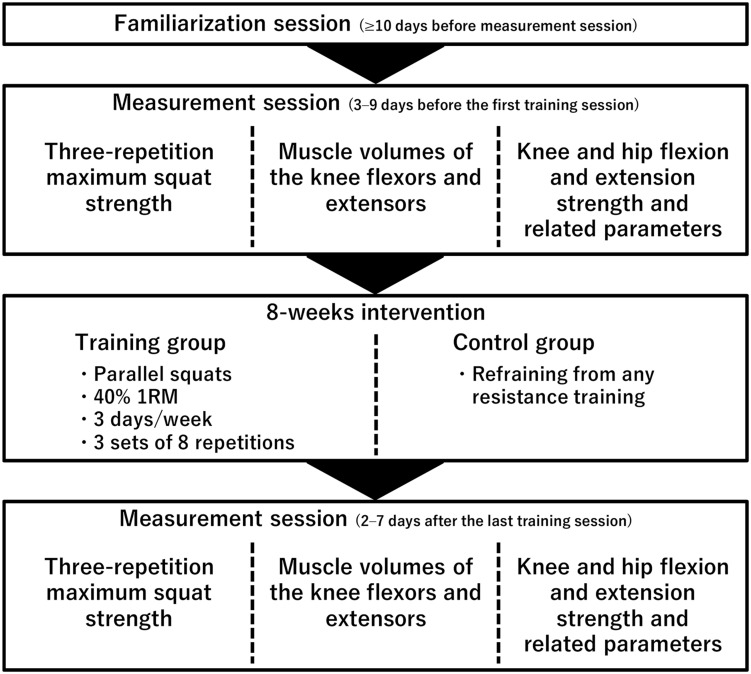
Schema of experimental procedures.

A schema of the experimental procedures is shown in Figure 1. The participants completed a familiarization session and a measurement session before (3–9 days before the first training session) and after (2–7 days after the last training session) the 8-week training or control period. In the familiarization session (≥10 days before the measurement session before the intervention), the participants performed maximal voluntary contraction (MVC) of isometric knee and hip flexion and extension. In the measurement sessions before and after the 8-week training or control period, three-repetition maximum squat strength (3RM), muscle volume, and other parameters were measured on different days. To safely assess their 1RM squat strength, 3RM was measured using a free-weight barbell and 1RM was estimated as follows: 1RM = 3RM/0.93 (kg) (Sheppard and Triplett, 2015). For parameters other than 3RM and muscle volume, the evoked quadriceps femoris twitch responses were determined first, followed by measurements of isometric peak torque of the knee flexors (KF) and KE and the hip flexors (HF) and extensors (HE) during MVC in random order. When measuring the peak torque of KE, voluntary activation was also evaluated. During these measurements, surface electromyography (EMG) signals of RF, the vastus lateralis, and medialis muscles (VL and VM) and the long head of BF were determined. The torque and EMG data were stored at a sampling frequency of 4 kHz on a personal computer using LabChart software (v8.1.5, ADInstruments, Australia) after 16-bit analog-to-digital conversion (PowerLab16/35, ADInstruments, Australia) to allow synchronization of the data where appropriate. Both before and after the 8-week training/control period, the muscle volume was measured before or at least 2 days after the other elements, and the 3RM measurement was conducted at least 3 days apart from the torque measurements. Measurements other than the 3RM measurements were recorded for the right leg only.

### Training

The participants of the training group performed parallel squats at 40% 1RM (i.e., low intensity), 3 days per week with 1–2 days of rest between sessions for 8 weeks. The training consisted of 3 sets of 8 repetitions with 3-min rest between sets. They were instructed to stand equally on both legs on the floor with their feet shoulder-width apart and angled outward at ~20–30° and to avoid moving their knee position back and forth as much as possible. After a warm-up, the participants performed parallel squats consisting of flexion (4 s) and extension (4 s) from the standing posture with the aid of a metronome. This speed was set to be slower than that in the study by Usui et al. (2016) (3 s), and is considered slow in accordance with a previous systematic review (Davies et al., 2017) that defined fast, moderate, and slow speeds as ≤ 1, 1–2, and > 2 s, respectively, for concentric/eccentric actions. Parallel squat depth was defined in advance as the position in which the thigh was parallel to the floor. The depth was limited by a tense rope with a bell set at the height of each participant’s squat depth, and real-time feedback was provided throughout the exercises by a display showing a video picture from a sagittal plane obtained by a video camera. On the first day of the third, fifth, and seventh weeks, the 3RM measurement was repeated before performing the training session to determine the next training intensity. For this purpose, a sufficient interval between the 3RM measurement and the training session was established to avoid any effect of fatigue on the training session. The participants of the control group were asked to refrain from any resistance training during the study period. In addition, all of the participants were requested to conduct their routine daily activities except performing squat training and to eat as usual during this period.

### 3RM Measurements

3RM was measured after several submaximal squat exercises at light load as a warm-up. Before the intervention, the initial load was each participant’s body mass. On the first day of the third, fifth, and seventh weeks, and after the intervention, the initial load was the previous 3RM for each participant. The load was then increased until the participants could successfully lower and raise the bar 3 times in the correct posture and speed (2 s for concentric/eccentric actions) but failed to achieve a fourth repetition. The increase in load was maintained to 5 times or less, with 5 min rest between attempts.

### EMG Electrode Placement

Of the four muscle groups, electrical nerve stimulation could be applied only to the KE to observe the neuromuscular adaptations of the KE induced by training. Hence, an EMG system (Bagnoli 8 EMG System, DELSYS, United States) was used to record EMG signals of the KE. Pre-amplified bipolar active surface EMG electrodes (electrode shape: parallel-bar, size: 1 mm width × 10 mm length, inter-electrode distance: 10 mm; DE-2.1, DELSYS, United States) with band-pass filtering between 20 and 450 Hz were placed on the bellies of each muscle along the fascicle direction after preparing the skin by shaving, abrasion with sandpaper, and cleaning with alcohol. The muscle belly and fascicle longitudinal directions were confirmed using an ultrasound apparatus (ACUSON S2000, Siemens Medical Solutions, United States). The EMG electrodes for the RF, VL and BF were located at 50% and for the VM, at 30% of the thigh length determined from the popliteal crease to the greater trochanter. A reference electrode was placed over the left lateral malleolus.

### Peak-to-Peak Compound Muscle Action Potential Amplitude (Mmax) Measurements

The quadriceps femoris twitch responses were obtained using a constant-current variable voltage stimulator (DS7AH, Digitimer Ltd., United Kingdom) with a controller (SEN-3401, Nihon Kohden, Japan) before the measurements of peak torque. A cathode (2 × 2 cm) and anode (4 × 5 cm) were placed in the femoral triangle and midway between the superior aspect of the greater trochanter and inferior border of the iliac crest, respectively, to percutaneously stimulate the femoral nerve with rectangular pulses of 200 μs. The stimulus intensity was gradually increased until a plateau in the twitch torque was reached. Afterward, supramaximal stimulus intensity was set at the electrical current calculated by multiplying the stimulus intensity by 1.2 for experimental measurements to ensure maximal muscle activation (Cresswell et al., 1995; Miyamoto et al., 2009). Resting-evoked twitch responses were obtained 5 times every 10 s to determine the Mmax of RF, VL, and VM. The Mmax values of each muscle were averaged for further analyses.

### Muscle Strength Measurements

A dynamometer (CON-TREX MJ, Physiomed, Germany) was used to measure the peak torque. Participants were instructed to sit on the reclining seat of the dynamometer with the hip at 80° and the knee at 90° flexion (anatomical position = 0°) when the peak torques of the KF, KE, and HF were measured and to lie in a supine position with the right hip and knee at 90° of flexion when the peak torque of the HE was measured. The pelvis and torso were secured to the reclining seat and dynamometer with non-elastic straps and/or a seat belt. Care was taken to adjust the centers of rotation of the knee/hip joints and dynamometer. An ankle strap for the measurements of the peak torques of KF and KE was attached slightly proximal to the lateral malleolus, and a pad to evaluate peak torque of HF and HE was positioned ~5 cm proximal from the upper border of the patella. The position of the participant in relation to the dynamometer and the strap or pad positions was carefully matched between the measurements before and after the 8-week training or control period using a scale attached to the dynamometer. After performing warm-up procedures consisting of submaximal contractions at intensities of ~30, 50, and 80% of MVC, the peak torque during MVC (for 3 s) was measured twice with a 1-min interval. If the difference between the two values of peak torque was > 10% of the higher torque, then additional contractions were performed until the difference between the highest and second highest values was less than 10% of the highest value; 1 min of rest was allowed between contractions. The average values of the first and second highest values of peak torque measurements were considered the peak torques of KF, KE, HF, and HE, respectively. The root mean square values of the EMG signals (RMS-EMGs) for the RF, VL, VM, and BF during MVC of the KE and RMS-EMG of the BF during MVC of the KF were evaluated over a 0.5-s period around the peak torque. These values in the selected tasks, in which the first and second highest values of peak torque were observed, were averaged. In addition, RMS-EMGs of the RF, VL, and VM during MVC of the KE were normalized to Mmax and RMS-EMG of the BF during MVC of the KE was normalized to that during MVC of the KF for further analyses.

### Voluntary Activation of KE Measurements

When measuring the peak torque of the KE, two supramaximal triplet stimulations at 100 Hz were interpolated ~ 3 s after the beginning and end of contraction to assess voluntary activation (Ema et al., 2018a). It was calculated using the following formula: Voluntary activation = [1 - (superimposed triplet torque/potentiated resting triplet torque)] × 100 (%). The two values of voluntary activation observed in the trials of the peak torque of KE were averaged for further analyses.

### Muscle Volume Measurements

As described in the Introduction, some of the hamstrings contributing to both hip extension and knee flexion (Frigo et al., 2010; Schache et al., 2010) and the RF contributing to both knee extension and hip flexion (Ema et al., 2018a, b) are bi-articular muscles, both of which are present in the thigh. Therefore, thigh muscle volume was measured using a 3.0-T whole-body magnetic resonance imaging scanner (MAGNETOM Verio or Skyra, Siemens Healthcare Diagnostics K. K., Germany). Transverse scans were performed with a conventional T1-weighted spin-echo sequence (repetition time, 500 ms; echo time, 8.2 ms; slice thickness, 10 mm; interspaced distance, 0 mm). Imaging was carried out on a field of view of 240 × 240 mm with a matrix size of 256 × 256. Within the device, participants lay in the supine position with their right heels placed on a handmade apparatus made of polystyrene foam. A series of transverse images of the relaxed right thigh were scanned. From the scanned images, the outlines of the BF (without distinction between the long and short heads), semitendinosus (ST), and semimembranosus (SM) (the KF) and RF, VL, VM, and VI (the KE) were digitized, and each cross-sectional area was measured once by a highly trained analyst using a personal computer with an image analysis software package (sliceOmatic ver 5.0 Rev-2d, TomoVision, Canada). This analyst had previous experience with similar measurements (Akagi et al., 2012, 2014). Non-contractile tissue, which was imaged in different tones from the contractile tissue, was excluded. Each muscle volume was automatically calculated by multiplying the sum of the cross-sectional area of each muscle group along their length by an interval of 10 mm using the image analysis software package.

### Habitual Physical Activity Measurements

The participants were instructed to wear a device (Active style Pro HJA-750C, Omron Health Care, Japan) to evaluate their habitual physical activity while performing activities of daily living for 11 days during the 8-week period, not including time spent bathing or sleeping. The data from days at which the participants wore the device for > 500 min/day as recorded in a diary were used for the analyses. As a result, the analysis period ranged from 4 to 11 days, which meets the criteria used in a previous study (Watanabe et al., 2020). The mean magnitude of physical activity was determined per day at three levels: light (<3.0 metabolic equivalents), moderate (3.0–5.9 metabolic equivalents), and vigorous (≥6.0 metabolic equivalents) (Bryan and Katzmarzyk, 2009; Lyden et al., 2011).

### Statistical Analyses

A three-way analysis of variance (ANOVA) with two within-group factors [time (before and after the intervention), region (KF, KE, HF, and HE)], and one between-group factor [group (the training and control groups)] was used to evaluate changes in peak torque induced by the squat training. A three-way ANOVA with two within-group factors [time (before and after the intervention), muscle (RF, VL, and VM)] and one between-group factor [group (the training and control groups)] was used for Mmax and RMS-EMGs normalized to Mmax, and a two-way ANOVA with one within-group factor [time (before and after the intervention)] and one between-group factor [group (the training and control groups)] was conducted for voluntary activation, i.e., normalized RMS-EMG of BF and 3RM, in order to assess the squat training-induced changes. Regarding evaluation of changes in each muscle volume after squat training, a three-way ANOVA with two within-group factors [time (before and after the intervention), muscle (BF, ST, and SM for KF), or (RF, VL, VI, and VM for KE)] was used. For assessing squat-training induced changes in the total muscle volume of KF or KE, two-way ANOVA with one within-group factor [time (before and after the intervention)] and one between-group factor [group (the training and control groups)] was used. In any case, when a significant interaction was observed, the Bonferroni multiple-comparison test was performed as additional ANOVA to investigate the effect of squat training on the corresponding variables. In addition, when a significant change in muscle strength and at least one muscle volume was found in the same muscle group (i.e., KF and/or KE), Pearson’s product-moment correlation coefficient was used to examine the relationships between the percent changes in muscle strength and volume from before to after squat training in the training group. To confirm the similarity in the magnitude of physical activity between the groups, two-way ANOVA with one within-group factor [level (light, moderate and vigorous)] and one between-group factor [group (the training and control groups)] was used.

Data are presented as means ± SDs. Statistical significance was set at *P* < 0.05. Statistical analyses were performed using statistical analysis software (SPSS 25.0, IBM, United States). The training effects on the variables that changed significantly after the intervention in the training group compared with the control group were estimated using an index of effect size (ES) for the pretest-posttest-control design in accordance with a previous study (Morris, 2008). The ES values were interpreted as < 0.20, 0.20–0.49, 0.50–0.79, and ≥ 0.80 for trivial, small, moderate, and large effects, respectively (Cohen, 1988, 1992).

## Results

### Habitual Physical Activity

The magnitudes of physical activity in the training group were 607 ± 64 min/day, 84 ± 14 min/day and 3 ± 6 min/day for light, moderate and vigorous levels, respectively. Those in the control group were 545 ± 108 min/day for light level, 83 ± 16 min/day for moderate level and 2 ± 1 min/day for vigorous level. The level × group interaction (*P* = 0.120) and the main effect of group (*P* = 0.112) were not significant.

### Peak Torque

**FIGURE 2:**
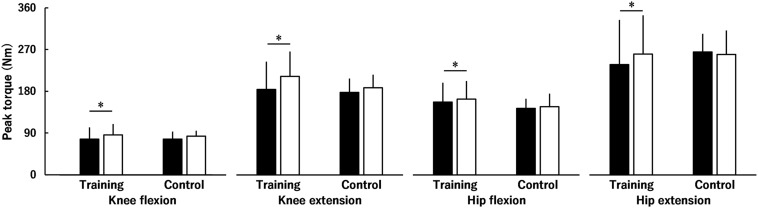
Peak torque of the knee and hip flexors and extensors before (black) and after (white) the intervention in the training and control groups. There was a significant group × time interaction with no significant group × time × region interaction, time × region interaction, or group × region interaction. * indicates a significant difference between the interventions. Data are presented as mean ± standard deviation.

Figure 2 shows the peak torque of the KF, KE, HF and HE before and after the intervention. There was a significant group × time interaction (*P* = 0.025) with no significant group × time × region (*P* = 0.068), time × region (*P* = 0.078), or group × region interactions (*P* = 0.337), and a significant main effect of region was found (*P* < 0.001; HE > KE > HF > KF). There was no significant group difference in the peak torques before (*P* = 0.905) or after (*P* = 0.434) the intervention. Peak torques of the KF, KE, HF, and HE in the training group significantly increased (*P* < 0.001) by 17.1 ± 21.9, 20.3 ± 22.3, 5.9 ± 9.3, and 15.8 ± 25.3%, respectively, but not in the control group (*P* = 0.317) after the intervention. The training effects on the peak torques of the KE (ES = 0.38) and HE (ES = 0.36) were small and those on the peak torques of the KF (ES = 0.13) and HF (ES = 0.09) were trivial.

### 3RM

The 3RM was 76.3 ± 15.9 kg in the training group and 78.5 ± 16.8 kg in the control group before the intervention and 100.0 ± 17.0 kg in the training group and 81.7 ± 17.2 kg in the control group after the intervention. A significant group × time interaction (*P* < 0.001) was found. Although there was no significant difference in 3RM between the groups before the intervention (*P* = 0.734), 3RM was significantly higher in the training group than in the control group after the intervention (*P* = 0.015). In the training group, 3RM significantly increased (*P* < 0.001) by 32.8 ± 14.5% after the intervention, but the corresponding change was not significant in the control group (*P* = 0.108). The ES was 1.22 (large).

### Voluntary Activation and EMG Variables During MVC of KE

Voluntary activation of the KE was 80.3 ± 14.4% in the training group and 81.4 ± 15.1% in the control group before the intervention and 81.8 ± 10.8% in the training group and 86.0 ± 6.9% in the control group after the intervention. There was no significant group × time interaction (*P* = 0.518) or main effect of group (*P* = 0.200) or time (*P* = 0.554).

**FIGURE 3:**
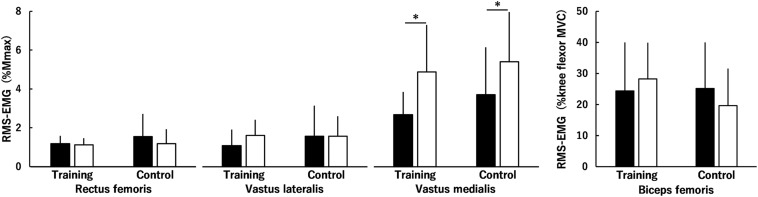
Root mean square values of surface electromyography signals (RMS-EMGs) of the rectus femoris muscle, the vastus lateralis and medialis muscles, and the biceps femoris muscle during maximal voluntary contractions (MVCs) of the knee extensors before (black) and after (white) the intervention in the training and control groups. The values of the rectus femoris muscle and the vastus lateralis and medialis muscles were normalized to the peak-to-peak compound muscle action potential amplitude (%Mmax), and those of the biceps femoris muscle were normalized to those during knee flexor MVC (%knee flexor MVC). Time × muscle × group, time × group and muscle × group interactions and a main effect of group were not significant. There was a significant time × muscle interaction. * indicates a significant difference between the intervention. Data are presented as mean ± standard deviation.

Time × muscle × group (*P* = 0.920), time × group (*P* = 0.122) and muscle × group (*P* = 0.594) interactions and the main effect of group (*P* = 0.318) were not significant for normalized RMS-EMGs of the RF, VL, and VM during MVC of the KE (Figure 3). There was a significant time × muscle interaction (*P* < 0.001) and a significantly higher normalized RMS-EMG after than before the intervention in the VM (*P* < 0.001) but not in the RF (*P* = 0.131) or VL (*P* = 0.127). Regarding normalized RMS-EMG of the BF during MVC of the KE, no significant time × group interaction (*P* = 0.196) or main effect of time (*P* = 0.816) or group (*P* = 0.375) was found (Figure 3).

### Muscle Volume

**FIGURE 4:**
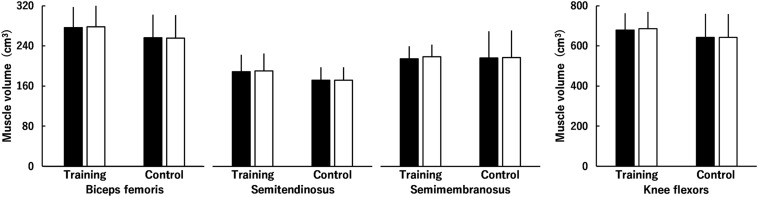
Muscle volume of the knee flexors (the biceps femoris muscle, semitendinosus muscle, and semimembranosus muscle) before (black) and after (white) the intervention in the training and control groups. Regarding muscle volume of the biceps femoris muscle, semitendinosus muscle, and semimembranosus muscle, time × muscle × group, time × group, and muscle × group interactions and main effects of time and group were not significant. Regarding total muscle volume of the knee flexors, time × group interaction and main effects of time and group were not significant. Data are presented as mean ± standard deviation.

**FIGURE 5:**
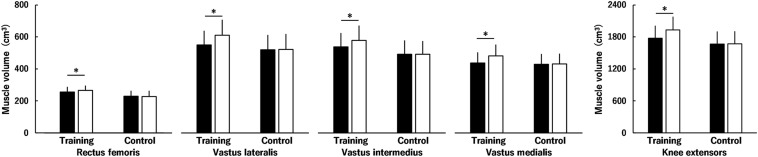
Muscle volume of the knee extensors (the rectus femoris muscle and the vastus lateralis, intermedius and medialis muscles) before (black) and after (white) the intervention in the training and control groups. Regarding muscle volume of the rectus femoris muscle and the vastus lateralis, intermedius, and medialis muscles, there was a significant time × muscle × group interaction. Regarding total muscle volume of the knee extensors, a time × group interaction was significant. * indicates a significant difference between the intervention. Data are presented as mean ± standard deviation.

Figures 4, 5 show muscle volume before and after the intervention. Regarding muscle volume of BF, ST, and SM (Figure 4), none of the interactions were significant (*P* = 0.081–0.891), and there were no main effects of time (*P* = 0.095) or group (*P* = 0.356). Similarly, the time × group interaction (*P* = 0.081) and main effects of time (*P* = 0.095) and group (*P* = 0.356) were not significant for the total muscle volume of KF (Figure 4). Regarding the muscle volume of RF, VL, VI, and VM (Figure 5), there was a significant second-order interaction (time × muscle × group: *P* < 0.001). No significant differences between the groups were found in each muscle volume before the intervention (RF, *P* = 0.059; VL, *P* = 0.443; VI, *P* = 0.201; VM, *P* = 0.780). Muscle volume of the RF, VL, VI and VM significantly increased by 3.8 ± 3.4, 11.2 ± 3.9, 7.5 ± 4.4, and 10.5 ± 4.3% in the training group (all *P* < 0.001), respectively, whereas significant changes were not found in the control group (RF, *P* = 0.775; VL, *P* = 0.732; VI, *P* = 0.995; VM, *P* = 0.575). For the total muscle volume of the KE (Figure 5), a time × group interaction was significant (*P* < 0.001). There was no significant difference between the groups (*P* = 0.265) before the intervention. In the training group, a significant increase in the total muscle volume of KE was found (8.9 ± 3.7%, *P* < 0.001), however, the corresponding difference in the control group was not significant (*P* = 0.806). The training effects on muscle volume of RF (ES = 0.28) and VI (ES = 0.45) were small, and those on muscle volume of the VL (ES = 0.64) and VM (ES = 0.64) and total muscle volume of KE (ES = 0.63) were moderate.

### Relationships Between Training-Induced Changes in Muscle Strength and Volume of KE

In this study, the training-induced increase in the peak torque of the KE and each muscle volume and total muscle volume of the KE were significant. On examining the relationships between the percentage changes before and after the intervention for the training group, the peak torque of the KE was significantly correlated with the muscle volume of the VL (*r* = 0.591, *P* = 0.043), VM (*r* = 0.647, *P* = 0.023), and the total muscle volume of the KE (*r* = 0.583, *P* = 0.047), but not with RF (*r* = 0.214, *P* = 0.505) or VI (*r* = 0.468, *P* = 0.125).

## Discussion

The main finding of the current study was that knee and hip flexion and extension strength improved after the 8-week low-intensity squat training at slow speed, supporting our hypothesis. However, the training effects on knee and hip flexion strength were limited compared with those on knee and hip extension strength.

Muscle hypertrophy and neuromuscular adaptations were evaluated in addition to muscle strength of the KE. The training-induced muscle hypertrophy was significant in the KE (Figure 5) and was more prominent for the VL and VM (ES = 0.64: moderate) than for the RF and VI (ES = 0.28 and 0.45: small). Regarding the percentage changes after the intervention for the training group, the percentage change in peak torque of the KE was significantly correlated with the percent changes in muscle volume of the VL and VM and total muscle volume of the KE but not with those in muscle volume of the RF or VI. In contrast, normalized RMS-EMGs of the RF and VL and the voluntary activation of the KE during MVC of the KE did not change after the squat training (Figure 3). Furthermore, normalized RMS-EMG of the VM during MVC of the KE increased after the intervention both for the training and control groups (Figure 3), although the reason for its increase in the control group cannot be ascertained. Thus, no training-induced neuromuscular adaptations of KE were found in the current study. Considering these results and the fact that muscle volume is a major determinant of joint torque in the KE (Akagi et al., 2012), the squat training-induced increase in knee extension strength can be influenced by the increase in the muscle volume of the KE, especially the VL and VM.

For HE, no training-induced hypertrophy of the hamstrings (the increase in total muscle volume of BF, ST, and SM) was found (Figure 4). However, in a recent study on the effect of 10-week high-intensity squat training at different depths (full or half squat) on lower limb muscle volume (Kubo et al., 2019), the training-induced rate of increase in the gluteus maximus muscle partially contributing to hip extension in the training group (6.7% for full and 2.2% for half) was reported to be comparable with that of the KE (4.9% for full and 4.6% for half). Hence, although we did not evaluate gluteus maximus muscle hypertrophy, we speculate that this hypertrophy affected the increased peak torque of the HE after squat training in the current study.

Both peak torques of the KF and HF were significantly increased following squat training (Figure 2), with trivial effects. In other words, the training effects on the peak torques of the KF and HF were less than those on the peak torques of the KE and HE. For the KF, significant hypertrophy was not found in any muscle, which is consistent with a recent study (Kubo et al., 2019). Previous studies showed that the activation levels of the hamstrings were lower than those of some of the KE during squat exercises as per EMG (Ema et al., 2017) or T-2 weighted MRI findings (Sugisaki et al., 2014). Sugisaki et al. (2014) suggested that a lack of change in the lengths of the hamstring muscles during squat exercises partially explains the weak activation of the hamstrings. Thus, it is considered that the hamstrings are used less than KE during squat exercises, as indicated by the previous study (Sugisaki et al., 2014), and this affected the current results of the difference in the muscle volumes of the KF (Figure 4) and KE (Figure 5). In other words, as suggested previously (Kubo et al., 2019), squat training appears to be insufficient to induce hypertrophy of the KF, likely resulting in the trivial training effect on the peak torque of the KF in the current study. Regarding the HF, training-induced hypertrophy of the RF, which is one of the HF, was found, but the rate of increase in the muscle volume of the RF (3.8%, ES = 0.28) was lower than that in the muscle volume of the vasti (7.5–11.2%, ES = 0.45–0.64). Based on these results, the training-related increase in the peak torque of the HF was not as pronounced as that of the KE.

In the current study, neuromuscular adaptations of the KF, HF, and HE were not investigated due to methodological limitations. Generally, it has been suggested that neuromuscular adaptations, rather than muscle hypertrophy, account for strength and power gains during the first few weeks of resistance training (Moritani and Devries, 1979; Akima et al., 1999; Laurentino et al., 2012). However, in the current study, neuromuscular adaptations did not occur even in the KE that had the largest training effect (ES = 0.38) on the peak torque of the four muscle groups. Hence, it is natural to think that neuromuscular adaptations may not have occurred not only in KE but also in the other muscle groups. Given the training specificity, the inconsistency of the movement during squat training with the single-joint movements when each muscle strength was measured may have been a factor in the absence of neuromuscular adaptations.

As mentioned previously, we interpreted the current results of the training-induced increases in knee and hip flexion and extension strength mainly using the results of muscle volume. However, when examining correlations between exercise-induced changes in muscle size and strength, these analyses are completed on groups designed to increase both muscle size and strength and also appear to be primarily correlating the error/random biological variability with muscle size with the error/random biological variability in muscle strength (Dankel et al., 2018). In other words, there is still no available evidence supporting the claim that changes in muscle size lead to changes in strength (Loenneke et al., 2019), and this aspect needs to be examined.

Usui et al. (2016) used an 8-week 50% 1RM squat training protocol at slow speed (3 s for concentric/eccentric actions) and found training-induced increase in strength of the HE but not of the KE. Our results were consistent with the previous ones on the peak torque of the HE but not of the KE. The squat training protocols of the current (4 s for concentric/eccentric actions) and previous studies were both performed at slow speeds, but there was a difference of 1 s between them. In addition, based on a systematic review (Raymond et al., 2013), 50% 1RM is considered to be a moderate-intensity protocol, while 40% 1RM, low-intensity one. The slower speed and lower intensity in our study (4 s and 40% 1RM) than those in the previous study (3 s and 50% 1RM) may have affected the difference in the training effect on the peak torque of the KE. On the other hand, the rate of increase in the peak torque of the HE was comparable between the current (15.8%) and previous (18%) studies, suggesting the effectiveness of low- or moderate-intensity squat training at slow speed on hip extensor strength.

The training effect on 3RM (ES = 1.22) was larger than that on each peak torque (ES = 0.09–0.38) in the current study. Furthermore, the training effects on the peak torque of the KE after 4 weeks of knee extension training and on the peak torque of the HF after 4 weeks of hip flexion training in a previous study (ES = 1.00–1.20) (Ema et al., 2018a) were also larger than the current ES values for the peak torque of the KE (ES = 0.38) and of HF (ES = 0.09), although there was a slight difference in the ES calculation method between the studies. These findings indicate the training specificity, however, we should also consider efficiency, whereby one type of training protocol increases the strength of several muscles simultaneously. Considering the larger training effects on the muscle volume of some of the KE compared with those on the muscle strength in the current study, squat training may be more effective for muscle hypertrophy than for increasing muscle strength for a wide range of individuals.

In summary, the current study investigated whether knee and hip flexion and extension strength increased after an 8-week low-intensity squat training program at slow speed. There was a significant training-induced increase in muscle strength. The training-related effect sizes of knee and hip flexion strength were smaller than those of knee and hip extension strength. These results suggest that the squat training protocol used here is useful for increasing both knee and hip flexion and extension strength, with greater training effects on extension strength than on flexion strength. Regarding the KE, a significant increase in muscle volume, especially of the VL and VM, was found after squat training, without neuromuscular adaptations. In addition, the increase in muscle volume significantly correlated with the training-induced increase in the peak torque of the KE. Thus, muscle hypertrophy may affect the improvement in muscle strength, at least for the KE, after 8-week low-intensity squat training at slow speed.

## Data Availability Statement

The raw data supporting the conclusions of this article will be made available by the authors, without undue reservation, to any qualified researcher.

## Ethics Statement

The studies involving human participants were reviewed and approved by the Ethics Committee of the Shibaura Institute of Technology. The participants provided their written informed consent to participate in this study.

## Author Contributions

RAk, SS, RAn, RE, and KH conceived and designed the experiments. RAk, SS, NH, NI, HT, RAn, and KH performed the experiments. RAk and SS analyzed the data. RAk drafted the manuscript and prepared the tables and figures. All authors interpreted the results of the research, edited, critically revised, approved the final version of the manuscript, and have agreed to be accountable for all aspects of the work related to its accuracy and integrity.

## Conflict of Interest

The authors declare that the research was conducted in the absence of any commercial or financial relationships that could be construed as a potential conflict of interest.
